# Prevalence of Vitamin K2 Deficiency and Its Association with Coronary Artery Disease: A Case–Control Study

**DOI:** 10.3390/diseases13010012

**Published:** 2025-01-11

**Authors:** Sameh A. Ahmed, Abdulaziz A. Yar, Anas M. Ghaith, Rayan N. Alahmadi, Faisal A. Almaleki, Hassan S. Alahmadi, Waleed H. Almaramhy, Ahmed M. Alsaedi, Man K. Alraddadi, Hussein M. Ismail

**Affiliations:** 1 Department of Pharmacognosy and Pharmaceutical Chemistry, College of Pharmacy, Taibah University, Madinah 42353, Saudi Arabia; 2 Department of Internal Medicine, College of Medicine, Taibah University, Madinah 42353, Saudi Arabiahusseinismail1@med.suez.edu.eg (H.M.I.); 3 Department of Cardiology, College of Medicine, Suez Canal University, Ismailia 41522, Egypt

**Keywords:** coronary artery disease, vitamin K2, menaquinone-4, menaquinone-7

## Abstract

Background/Objectives: Vitamin K2 analogs are associated with decreased vascular calcification, which may provide protective benefits for individuals with coronary artery disease (CAD) by stimulating anti-calcific proteins like matrix Gla protein and adjusting innate immune responses. This study addresses a significant gap in understanding the association between serum levels of vitamin K2 analogs in different CAD types and examines their correlations with clinical risk parameters in CAD patients. Methods: This case–control study enrolled CAD patients and healthy controls to assess and compare serum concentrations of two vitamin K2 analogs including menaquinone-4 (MK-4) and menaquinone-7 (MK-7) via ultra-performance liquid chromatography with tandem mass spectrometry (UPLC-MS/MS). CAD risk factors were evaluated and related to serum levels of vitamin K2 analogs. The CAD group was further subdivided into stable angina, STEMI, NSTEMI, and unstable angina groups to investigate potential differences in vitamin K2 analog levels. Results: Patients experiencing acute coronary syndrome exhibited notably reduced serum levels of MK-4 and MK-7 (1.61 ± 0.66, and 1.64 ± 0.59 ng/mL, respectively) in comparison to the control group (2.29 ± 0.54, and 2.16 ± 0.46 ng/mL, respectively), with MK-4 and MK-7 displaying stronger associations with CAD risk indicators. Notable variations in vitamin K2 analog levels were found between CAD patients and control groups (*p* < 0.001). Unstable angina patients showed the lowest serum levels of MK-4 and MK-7. Conclusions: The present study demonstrated a higher prevalence rate of vitamin K2 deficiency among patients with CAD. The most pronounced decrease in MK-4 and MK-7 was observed in unstable angina patients. Moreover, these outcomes indicate the imperative requirement for an integrative approach that incorporates metabolic, lipid, and vitamin K2-related pathways in the risk stratification and management of CAD.

## 1. Introduction

Cardiovascular disease (CVD) has a significant impact on global mortality rates, including in Saudi Arabia [1]. Though there have been improvements in combating and dealing with the factors leading to CVD, the death rate is still high [2]. Vascular calcification, a hallmark of CAD, contributes to arterial stiffness, reduced coronary blood flow, and increased risk of adverse cardiovascular events [3,4,5]. The key factor in improving outcomes for CAD patients is to promptly prevent and intervene in vascular calcification using thorough risk factor management strategies [6]. This underscores the importance of identifying novel and modifiable risk factors to improve the prevention and management of CAD.

Recent research has revealed potential benefits of vitamin K2 analogs for CAD. Vitamin K2, also referred to as menaquinone (MK), is a fat-soluble vitamin present in fermented foods and synthesized by bacteria in the colon. MK’s form includes a variety of chemical compounds identified as MK1 to MK10 [7]. Vitamin K2 analogs primarily aid in the γ -carboxylation of various vitamin K-dependent proteins [8]. Nevertheless, vitamin K-dependent proteins go beyond the clotting factors. An important instance is the matrix Gla protein (MGP), which is a small protein found outside the cells. Vascular smooth muscle cells are the main source of production of MGP. It goes through two different types of post-translational modifications to fully mature: γ-glutamate carboxylation and serine phosphorylation, both of which require vitamin K [9]. Deficiencies in vitamin K2 have been linked to impaired carboxylation of MGP, promoting vascular calcification and CAD progression. Additionally, vitamin K2 analogs exhibit anti-inflammatory and antioxidant properties that may further contribute to their cardioprotective effects [10].

In addition, it is crucial to emphasize the significance of other vitamin K-dependent proteins in heart health, such as bone Gla protein, also known as osteocalcin (OC) [11]. This protein plays several roles in preserving heart health, especially in controlling vascular calcification. The complex connection between OC and heart health is shown through their ability to increase the production of adiponectin, a protein that blocks the buildup of calcium in smooth muscle cells of blood vessels, helping to prevent vascular calcification. OC carboxylation with vitamin K is essential for enhancing bone and cardiovascular health, particularly in improving outcomes of CAD [12]. Carboxylation is a vital post-translational modification of osteocalcin that relies on vitamin K to convert specific glutamic acid residues into gamma-carboxyglutamic acid (Gla) residues. Although OC has mainly been linked to bone health in the past, new studies indicate potential effects on heart health, like coronary artery disease (CAD) [13]. Properly carboxylated osteocalcin may provide benefits for bone mineralization that extend beyond the skeletal system [14,15].

Moreover, recent studies have discovered anti-inflammatory properties in vitamin K2 analogs, which could lower the risk of developing coronary artery disease. Research indicates that vitamin K2 analogs could reduce inflammation in the walls of arteries, a key factor in the progression of coronary artery disease [16]. Vitamin K2 analogs also act as antioxidants, reducing the risk of artery damage by combating oxidative stress. The defensive role of antioxidants protects the inner linings of the arteries and improves their optimal performance [17,18]. Research in epidemiology has discovered connections between increased consumption of vitamin K2 analogs in diets and a decreased chance of CAD or cardiovascular incidents. Although not proving a direct connection, these findings imply possible cardiovascular benefits from sufficient vitamin K2 analog levels [19].

The primary goal of this study is to investigate the relationship between serum levels of MK-4 and MK-7 and CAD risk factors. To achieve this, we compare these vitamin K2 analogs in CAD patients and healthy controls, analyze their correlations with lipid profiles and other clinical parameters, and assess their potential role in CAD pathogenesis. This research aims to fill a critical gap in understanding the role of vitamin K2 in cardiovascular health and to explore its potential as a biomarker for CAD risk assessment.

## 2. Materials and Methods

### 2.1. Study Population

This study enrolled 99 patients treated for CAD at Medina Cardiac Centre (MCC), Medina, KSA, and 81 subjects who had never been treated for any type of ischemic heart disease between July 2021 and October 2022. Patients with a history of recent or past CAD were included in this study. According to the inclusion criteria, Saudi patients with CAD (both males and females), with an average age of 57.3 years, were classified into the following categories: stable angina, ST elevation MI (STEMI), non-ST elevation MI (NSTEMI), and unstable angina. Patients with acute infectious diseases, malignancy, and acute or chronic liver disease were excluded from this study. The control group included age- and gender-matched subjects with no history of CAD. These participants were recruited from the same population as the case. We excluded CAD in the controls based on clinical history and absence of cardiovascular symptoms and normal findings on non-invasive diagnostic tests such as ECGs and echocardiograms when clinically indicated. Anyone who had suffered from a heart attack or had chest pain was not part of the study.

### 2.2. Data Collection Methods

The protocol was approved by the Research Ethics Committee of the College of Medicine at Taibah University, Al-Medina Al-Munawara, Saudi Arabia (IRB00010413). All participants were volunteers and provided informed consent. The research was conducted in accordance with the Helsinki Declaration and Saudi Arabian research ethics standards. The blood samples were collected from patients with CAD at MCC by the medical staff. The blood samples were collected from CAD patients at the time of admission to the hospital, prior to any therapeutic intervention. A portion of the blood samples used to measure the serum levels of MK4 and MK-7 was immediately centrifuged on site at 2000× *g* for 10 min at 4 °C and then stored at −70 °C until analysis. Serum samples were thawed at 25 °C and analyzed using high-performance liquid chromatography at the College of Pharmacy, Taibah University laboratories. We performed a general assessment, including blood pressure and pulse rate measurements, as well as a heart examination. Additionally, routine laboratory investigations, such as the assessment of serum lipids and uric acid, were performed at the MCC; blood samples were sent to the laboratory to assess vitamin K2 analog levels including MK-4 and MK-7 measured using ultra-performance liquid chromatography (UPLC) with tandem mass spectrometry [20]. The electrocardiogram and echocardiography findings were recorded.

### 2.3. Data Analysis

The SPSS software (version 21; SPSS Inc., Chicago, IL, USA) was used for data analysis. The results of the reactive species levels are expressed as the mean ± standard error of the mean. At first, the variables in the study were analyzed and we conducted normality tests, such as the Shapiro–Wilk test, to determine their distribution. Based on the results, the variables were found to be normally distributed. Consequently, the results are reported as mean ± standard error of the mean (SEM) and compared across groups using the independent t-test. The *p*-value was used to determine significance, with *p* > 0.05, *p* < 0.05, and *p* < 0.001 regarded as non-significant, significant, and highly significant, respectively. Pearson’s correlation coefficient was used to express the correlation between vitamin K2 analog serum levels in CAD patients and hematological, lipid profile, and clinical parameters.

## 3. Results

Table 1 presents a thorough comparison of demographic and health-related factors between a group of 99 CAD patients and a control group of 81 individuals, highlighting significant disparities in coronary artery disease risk factors. Although most individuals in both groups are over 45 years old based on the age distribution, there is no statistical significance in the difference. The gender breakdown is similar, with males making up the larger portion in both categories. The history of diabetes and hypertension shows no statistical significance between CAD patients and the control group. Smoking prevalence is higher among CAD patients (36.37%) compared to controls (24.70%), but the difference is not statistically significant. However, BMI stands out as a crucial distinguishing factor. Patients with coronary artery disease (CAD) have a higher prevalence of obesity (BMI ≥ 30), at 39.40% compared to 22.22% in the control group, and a lower prevalence of a healthy weight (BMI < 25), at 18.18% compared to 37.04% in controls, with both differences being statistically significant (*p* ≤ 0.05). Both groups show a similar distribution of the BMI category of 25–29.9 with no significant variance. These results indicate that increased BMI, as well as diabetes and hypertension, are important risk factors linked to CAD, while factors such as age, gender, and smoking status do not display statistically significant relationships in this study.

Table 2 displays an extensive comparison of hematological tests, lipid profiles, and clinical characteristics between CAD patients and a control group. Notable variations were noted in the parameters related to coronary artery disease (CAD) and cardiovascular health, among the two groups. In CAD patients, the average troponin level was 11.59 ± 7.40 ng/mL, while the control group had undetectable levels, with a *p*-value of ≤0.001 which was highly significant. Patients with CAD had a mean LV ejection fraction of 48.0 ± 9.96, which was notably lower. The average glucose levels were significantly elevated in individuals with CAD, measuring 164.76 ± 90.83 mg/dL, in contrast to 123.46 ± 22.17 mg/dL in the control group, with a highly significant *p*-value of ≤0.001. Values for parameters including hemoglobin (Hb), hematocrit (HCT), red blood cells (RBCs), mean corpuscular volume (MCV), mean corpuscular hemoglobin (MCH), mean corpuscular hemoglobin concentration (MCHC), and total white blood cells (WBCs) did not differ significantly between CAD patients and the control group, as indicated by non-significant *p*-values. There was a notable variation in platelet levels between CAD patients and the control group, with CAD patients having an average platelet count of 275.01 ± 90.48 × 10^3^/uL, higher than the control group’s 221.17 ± 27.17 × 10^3^/uL, with a *p*-value less than 0.001. CAD patients had notably higher total cholesterol and LDL cholesterol levels compared to controls in the lipid profile, with *p*-values of ≤0.001 for both. CAD patients had a significantly lower level of HDL cholesterol (0.92 ± 0.26 mmol/L) compared to controls (1.18 ± 0.11 mmol/L), with a *p*-value of ≤0.05. Triglyceride levels did not show a significant difference between the two groups. Systolic and diastolic blood pressure readings were higher in CAD patients (154.41 ± 19.45 mmHg and 101.82 ± 12.24 mmHg, respectively) compared to the control group (118.43 ± 11.98 mmHg and 78.11 ± 10.54 mmHg). However, these differences were not statistically significant.

We conducted a post hoc power analysis for our primary outcomes, the serum levels of MK-4 and MK-7. Using the observed effect sizes of 1.95 for MK-4 and 1.90 for MK-7, along with our sample sizes of 99 CAD patients and 81 controls, the statistical power was calculated to be 100% at a significance level of 0.05. These results confirm that our study was adequately powered to detect the significant differences observed. As shown in Table 3, the serum levels of vitamin K2 analogs, including MK-4 and MK-7, in CAD patients were measured and compared to a control group. The mean MK-4 levels were significantly reduced in CAD patients, averaging 1.60 ± 0.66 ng/mL, compared to 2.79 ± 0.54 ng/mL in the control group, with a highly significant *p*-value of ≤0.001. Additionally, MK-7 levels in CAD patients were 1.63 ± 0.60 ng/mL, markedly lower than the 2.66 ± 0.46 ng/mL observed in the control group, also with a *p*-value of ≤0.001.

**Figure 1:**
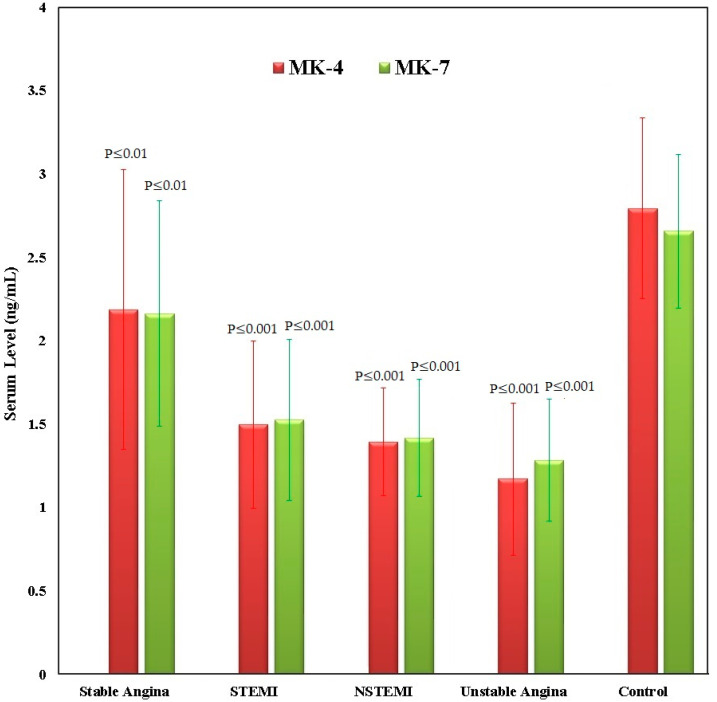
Serum levels of vitamin K2 analogs **±** SEM in CAD patients (Stable angina (n = 24), STEMI (n = 48), NSTEMI (n = 17), and unstable angina (n = 10)) groups and control (n = 81) group.

Figure 1 shows the serum levels of vitamin K2 analogs, MK-4 and MK-7, across five groups: stable angina, STEMI (n = 48), NSTEMI (n = 17), unstable angina (n = 10) and controls (n = 81). The control group shows the highest serum levels of MK-4 and MK-7, indicating a greater vitamin K2 analog status in healthy people. In CAD conditions, patients with stable angina show comparatively higher levels of MK-4 and MK-7 than patients in other CAD categories, yet these levels remain lower than those of the control group (*p* ≤ 0.01). Patients experiencing STEMI and NSTEMI show significantly lower levels of MK-4 and MK-7 when compared to controls (*p* ≤ 0.001). Patients with unstable angina show the lowest levels of these vitamin K2 analogs, with notable deficiencies compared to the controls (*p* ≤ 0.001). These results indicate a significant link between lower vitamin K2 analog levels and the severity of CAD, reinforcing the possible protective function of vitamin K2 analogs in managing CAD.

**Figure 2:**
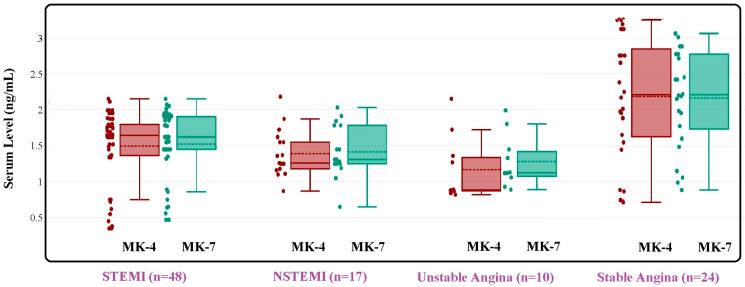
Box and whisker plot for serum levels of vitamin K2 analogs ± SEM in CAD patients (stable angina (n = 24), STEMI (n = 48), NSTEMI (n = 17), unstable angina (n = 10)) groups.

The box and whisker plot in Figure 2 depicts the serum levels of vitamin K2 analogs, MK-4 and MK-7, across four different groups of CAD patients: stable angina, STEMI, NSTEMI, and unstable angina. In the STEMI group, the MK-4 levels are significantly low, with a median of approximately 1.52 ng/mL and a narrower distribution. The MK-7 levels are even lower, with a median close to 1.48 ng/mL, showing a similar distribution pattern to MK-4 but with some lower outliers. In the NSTEMI group, the MK-4 levels are significantly reduced, with a median of around 1.42 ng/mL, showing broader variability, while the MK-7 levels are similar to MK-4, with a median of about 1.40 ng/mL. In the unstable angina group, the MK-4 levels are the lowest among all groups, with a median below 1.17 ng/mL, and the MK-7 levels also follow this trend, indicating a broader distribution but lower median levels. The stable angina group shows the highest median levels of vitamin K2 analogs among all groups. The MK-4 levels are slightly lower, with a median of around 2.19 ng/mL, and the MK-7 levels follow a similar pattern with a median of 2.16 ng/mL. These data suggest that patients with stable coronary artery disease maintain higher serum levels of vitamin K2 analogs compared to those with more acute conditions like STEMI, NSTEMI, and unstable angina.

**Figure 3:**
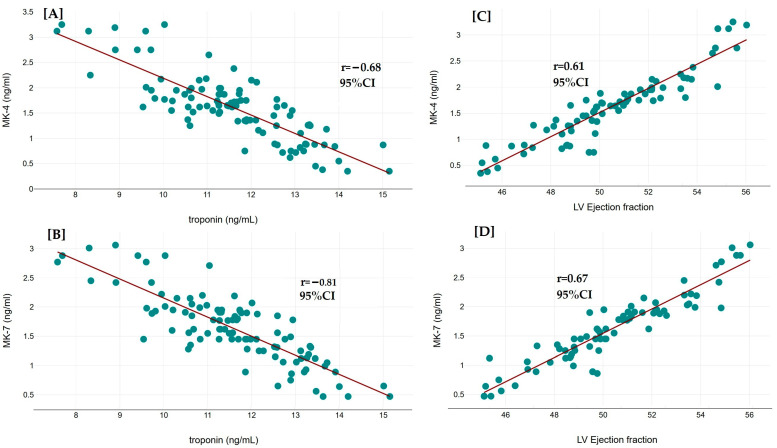
Scatterplots with regression lines for the relationship between serum troponin levels and MK-4 levels (**A**), serum troponin levels and MK-7 levels (**B**), LV ejection fraction and MK-4 levels (**C**), and LV ejection fraction and MK-7 levels (**D**).

The correlations between serum levels of vitamin K2 analogs (MK-4 and MK-7) and various hematological, lipid profile, and clinical parameters in CAD patients are presented in Table 4. The table provides the Pearson correlation coefficients (r values) for each parameter with MK-4 and MK-7 levels. Troponin, a critical marker of myocardial injury, shows a negative correlation with all two vitamin K2 analogs. Specifically, the correlation coefficients are −0.68 for MK-4 and −0.81 for MK-7. These negative correlations indicate that higher levels of vitamin K2 analogs are associated with lower troponin levels in CAD patients. The left ventricular EF exhibits positive correlations with MK-4 and MK-7, with r values of 0.61, and 0.67, respectively. This suggests that higher levels of vitamin K2 analogs are associated with better cardiac function, as indicated by a higher LV ejection fraction. Glucose levels show negative correlations with vitamin K2 analogs: −0.64 for MK-4 and −0.75 for MK-7. These results suggest that higher serum levels of vitamin K2 analogs are associated with lower glucose levels in CAD patients. For hemoglobin (Hb), hematocrit (HCT), red blood cells (RBCs), mean corpuscular volume (MCV), mean corpuscular hemoglobin (MCH), mean corpuscular hemoglobin concentration (MCHC), total white blood cells (WBCs), and platelets, the correlations with vitamin K2 analogs are generally weak and negative. The most notable correlations are observed with platelets, where the r values are −0.35 for MK-4 and −0.42 for MK-7, indicating a moderate negative relationship. In the lipid profile, total cholesterol, LDL cholesterol, and triglycerides exhibit negative correlations with all two vitamin K2 analogs. The strongest negative correlations are seen with total cholesterol (−0.71 with MK-4 and −0.77 with MK-7) and LDL cholesterol (−0.68 with MK-4 and −0.73 with MK-7). Conversely, HDL cholesterol, which is protective against cardiovascular disease, shows positive correlations with MK-4 (0.60) and MK-7 (0.69). We also conducted additional analyses to examine the correlations between MK-4, MK-7, and lipid profile parameters (total cholesterol, LDL cholesterol, HDL cholesterol, and triglycerides) in the combined cohort of CAD patients and controls. Our analysis revealed significant negative correlations between MK-4 and MK-7 levels with total cholesterol (*r* = −0.70, *p* < 0.001 and *r* = −0.76, *p* < 0.001, respectively) and LDL cholesterol (*r* = −0.67, *p* < 0.001 and *r* = −0.73, *p* < 0.001, respectively). Conversely, both MK-4 and MK-7 showed significant positive correlations with HDL cholesterol (*r* = 0.59, *p* < 0.001 and *r* = 0.68, *p* < 0.001, respectively). There was a weaker, non-significant correlation between MK-4, MK-7, and triglycerides in the combined cohort. Correlations in the combined cohort provide insights into the preventive role of MK-4 and MK-7, potentially highlighting how these analogs relate to lipid profiles and other risk factors across a spectrum of health conditions and diseases. As seen in Figure 3, the scatterplots show a negative correlation between serum troponin and MK-4 levels (as well as serum troponin and MK-7 levels). In the same way, the LV ejection fraction positively correlates with MK-4 levels and MK-7 levels.

## 4. Discussion

This study has shown that there are substantial differences in the main biomarkers and clinical characteristics between CAD patients and the control group; this supports the multifactorial nature of CAD and its wide-ranging influences on cardiovascular diseases [21]. It is important to emphasize the interplay of metabolic, hematological, and cardiovascular systems in CAD, which requires an integrative strategy for the assessment of risk factors, as well as their management [22]. Myocardial infarction or other heart diseases are usually associated with elevated troponin levels in these individuals, suggesting myocardial injury among them, while people without CAD have very low troponin levels [23]. Furthermore, significantly decreased LV ejection fraction among individuals with CAD implies compromised heart workings common in ischemic heart disease, while the normal LV ejection fraction observed within the control group also proves the absence of any cardiac dysfunction among individuals without any kind of coronary artery disease [24].

The incidence of elevated blood glucose levels in CAD patients may point towards possible conditions such as diabetes or impaired glucose tolerance, as there is a strong relation between hyperglycemia and CAD. These conditions raise questions about their relationship with cardiovascular diseases and why they need to be controlled for these reasons. Additionally, recent studies which employed the Mendelian randomness method have also discovered that people who have more vitamin K in their systems are less likely to suffer from diabetes mellitus [25], thereby suggesting a connection between vitamin K and metabolic health.

The lack of significant differences in hematological parameters between the two groups might imply that CAD does not directly influence these factors in this cohort. Although hemoglobin, hematocrit, RBC count, MCV, MCH, MCHC, and WBCs are essential for overall health, they seem to play a minimal role as far CAD is concerned in this study. On the contrary though, the significantly raised platelet counts seen in CAD patients indicate more active platelets which aggregate more, thus being very important for the pathogenesis of both atherosclerosis and thrombosis. This is consistent with what is known about how platelets contribute to the formation of plaques and the increased likelihood of thrombotic events, thus making it important to monitor their activity when managing patients with CAD [26].

The lipid profile results reaffirm the well-established association between dyslipidemia and CAD. Elevated total and LDL cholesterol levels in CAD patients highlight the need for lipid management to reduce cardiovascular risk [27]. Additionally, the lower HDL cholesterol levels observed in CAD patients further emphasize the inverse relationship between HDL cholesterol and CAD risk, reinforcing the necessity for comprehensive lipid management in preventing and treating CAD. The non-significant difference in triglyceride levels suggests that triglycerides may not be as strongly associated with CAD as other lipid parameters in this cohort.

Even though the higher blood pressure readings in the CAD patients did not reach statistical significance, the trend corresponds to the role of hypertension, which is widely regarded as one of the most prominent risks to the cardiovascular system. This suggests that management of blood pressure is still a major component in controlling CAD even if there were no statistically significant differences in this study [28]. A recent cohort study aimed at investigating the relationship between serum 25(OH) vitamin D, dp-ucMGP and blood pressure among older adults [29]. Low levels of vitamin D3 and its metabolites were reported in acute coronary syndrome patients [30]. The findings indicated that lower levels of vitamin D and vitamin K together contribute to increased systolic and diastolic blood pressure and an equal risk for developing hypertension. Cardiovascular health greatly depends on vitamin K analogs through their regulation on vascular calcifications as well as the preservation of arterial status [31]. The serum MK-4 and MK-7 levels were significantly different between CAD patients and controls, indicating that deficiencies in these vitamin K compounds might be linked with its pathogenesis [16].

MK-4, which is among the most significant forms of vitamin K2 analogs, activates proteins responsible for regulating bone and vascular health [32]. The marked reduction in MK-4 levels noted in CAD patients could indicate a deficiency in this type of vitamin K2 analog that prevents vascular calcification. In addition, CAD patients’ lower MK-4 levels might reflect lesser dietary intake of foods rich in MK-4 or its impaired formation from K1. Such deficiency would worsen vascular calcification since MK-4 inhibits blood vessel solidification through its action on MGP and other proteins requiring this micronutrient. This shows how MK-4 is crucial for heart-related well-being and how it may play a role in causing CAD.

MK-7, one of the forms of vitamin K2 analogs, is reported to have a longer half-life compared to other vitamin K2 analogs and therefore will be more available. MK-7 is critical for activating proteins that are responsible for regulating calcium metabolism and promoting vascular health [33]. Levels of MK-7 were significantly reduced in patients with CAD who may then suffer from possible MK-7 deficiency in the disease’s development or progression. This would explain why lower levels of this vitamin K2 analog are capable of adequately suppressing calcification in vessels. It could hence trigger albumin-atherosclerotic development, which is one major cause behind CAD. However, it should be noted that reduced intake via lower dietary sources or absorption issues among these individuals would lead to reduced levels of MK-7 within them.

The observed trend of decreased serum levels of vitamin K2 analogs in CAD patients, particularly those with stable angina, STEMI, NSTEMI, and unstable angina, compared to the control group, is notable. Within STEMIs and NSTEMIs, this reduction is at its peak, with them having the lowest concentration levels, especially MK-7. All vitamin K2 analogs primarily exhibit high concentrations within control participants. This finds resonance with various reports which have intimated that its vascular calcification inhibition properties plus inflammation mitigations make it play a cardio-protective role [16,31]. Out of available vitamin K2 analogs, MK-7 has shown the greatest difference in patient groups. Since there are significantly lowered levels of MK-7 in STEMI and NSTEMI patients, it can be speculated that this isoform plays a role during acute coronary syndrome genesis. The reduction in this nutrient among severe CAD cases may suggest that MK-7 is a more active compound than MK-4 and, with a longer half-life, is depleted because of acute myocardial injury.

New evidence indicates that adding vitamin K2 analogs to the diet could have a positive impact on treating CAD. Vitamin K2 analogs such as MK-7 and MK-4 are known for their ability to regulate the activation of MGP, which plays a role in preventing vascular calcification. Several studies have suggested that consuming vitamin K2 analog supplements may help to delay the progression of atherosclerosis and improve the flexibility of arteries by promoting the activation of MGP to decrease calcification [34]. Moreover, vitamin K2 analogs are thought to have properties that can combat inflammation and oxidative stress, which could potentially decrease subclinical inflammation, a known contributor to the onset of coronary artery disease. Recent studies are looking into the potential benefits of vitamin K2 analog supplementation in reducing cardiovascular events and enhancing heart health in high-risk populations, such as individuals with CAD [35]. With this increasing amount of research, vitamin K2 analog supplementation shows potential as an additional treatment option for CAD, but more large-scale trials are necessary to determine clear clinical guidelines. To the best of our knowledge, the current study is the first to directly measure circulating levels of MK-4 and MK-7 in CAD patients. Most previous research focuses on the effects of vitamin K2 supplementation or dietary intake rather than baseline serum levels. This distinction underscores a significant gap in the literature, which our study addresses by providing a novel perspective on the role of MK-4 and MK-7 in CAD.

There are limitations of the present study. Due to a lower sample size, the results may lack generalizability, which restricts our ability to draw robust conclusions about the relationship between vitamin K analog levels and CAD severity. Also, multiple regression analysis would better account for confounders and provide stronger conclusions. Therefore, further studies must be carried out with larger sample sizes to validate and expand upon these findings. The fact that this was a cross-sectional study means that these findings cannot be interpreted in a causal way, and future studies should be longitudinal. We also have measured serum MK-4 and MK-7 levels at baseline, not adjusting for dietary intake, supplementation, or treatment, which might have affected the results. Finally, although the controls underwent CAD exclusion with non-invasive methods, the lack of advanced imaging may dilute the robustness of CAD exclusion.

## 5. Conclusions

This research emphasizes the prevalence of vitamin K2 deficiency and the association with CAD. Our results indicate that these deficiencies are significantly associated with the severity of CAD and related risk factors. The interactions of vitamin K2 analogs with metabolic, hematological, and cardiovascular systems further underscores the complexity of CAD. The potential protective role of vitamin K2 analogs, especially MK-7, in preventing vascular calcification and supporting cardiovascular health is particularly noteworthy, suggesting the need for further exploration of nutritional interventions in CAD management. These findings reinforce the necessity of adopting an integrative approach that con-siders metabolic, lipid, and vitamin K2-related factors in CAD risk assessment and management, thereby contributing to more personalized and effective strategies for preventing and treating this widespread cardiovascular condition.

## Figures and Tables

**Table 1:** General characteristics of CAD patients and control groups.

Variables	CAD Patients (n = 99)	Control (n = 81)	*p*-Value
**Age (Mean ± SEM)**	53.6 ± 10.2 years	51.2 ± 9.8 years	0.092
**Gender**			
Male	80 (80.80%)	61 (75.30%)	0.171
Female	19 (19.20%)	20 (24.70%)	
**History of Diabetes**			
No	29 (29.29%)	13 (16.05%)	0.156
Yes	70 (70.71%)	68 (83.95%)	
**History of Hypertension**			
No	8 (8.09%)	15 (18.52%)	0.212
Yes	91 (91.91%)	66 (81.48%)	
**Smoking**			
No	63 (63.63%)	61 (75.30%)	0.132
Yes	36 (36.37%)	20 (24.70%)	
**BMI**			
<25	18 (18.18%)	30 (37.04%)	≤0.05
25–29.9	42 (42.42%)	33 (40.74%)	
≥30	39 (39.40%)	18 (22.22%)	

BMI, body mass index; CAD, coronary artery disease.

**Table 2:** The hematological tests, lipid profiles, and clinical characteristics between the CAD patients and control group.

Parameters	CAD Patients (n = 99) Mean ± SEM	Controls (n = 81) Mean ± SEM	*p*-Value *
Troponin (ng/mL)	11.59 ± 7.40	-	-
LV ejection fraction (%)	48.0 ± 9.96	-	-
Glucose Level (mg/dL)	164.76 ± 90.83	123.46 ± 22.17	≤0.001
Hb (g/dL)	12.86 ± 1.83	12.16 ± 0.86	0.129
HCT (%)	41.44 ± 5.04	36.07 ± 2.26	0.090
RBCs (10^6^/uL)	5.08 ± 0.76	4.71 ± 0.63	0.110
MCV (fL)	82.97 ± 6.87	80.92 ± 66	0.090
MCH (pg)	27.99 ± 3.74	27.12 ± 2.77	0.157
MCHC (g/dL)	33.90 ± 1.43	30.08 ± 1.77	0.095
Total WBCs (10^3^/uL)	10.73 ± 4.31	9.08 ± 1.24	0.105
Platelets (10^3^/uL)	275.01 ± 90.48	221.17 ± 27.17	<0.001
Total Cholesterol (mmol/L)	4.78 ± 1.33	3.10 ± 0.42	≤0.001
HDL-Cholesterol (mmol/L)	0.92 ± 0.26	1.18 ± 0.11	≤0.05
LDL-Cholesterol (mmol/L)	3.22 ± 1.15	1.85 ± 0.69	≤0.001
Triglycerides (mmol/L)	1.35 ± 0.79	1.24 ± 0.90	0.082
Systolic Blood Pressure (mmHg)	154.41 ± 19.45	118.43 ± 11.98	0.067
Diastolic Blood Pressure (mmHg)	101.82 ± 12.24	78.11 ± 10.54	0.086

* Calculated using Independent Samples *t*-Test. Hb, hemoglobin; HCT, hematocrit; HDL, high-density lipoproteins; LDL, low-density lipoproteins; MCH, mean corpuscular hemoglobin; MCHC, mean corpuscular hemoglobin concentration; MCV, mean corpuscular volume; RBCs, red blood cells.

**Table 3:** Serum levels of vitamin K2 analogs in the CAD patients and control group.

Vitamin K2 Analogs	CAD Patients (n = 99) Mean ± SEM	Controls (n = 81) Mean ± SEM	*p*-Value *
MK-4 (ng/mL)	1.60 ± 0.66	2.79 ± 0.54	≤0.001
MK-7 (ng/mL)	1.63 ± 0.60	2.66 ± 0.46	≤0.001

* Calculated using Independent Samples *t*-Test.

**Table 4:** Correlations of hematological tests, lipid profiles, and clinical parameters with serum levels of vitamin K2 analogs in CAD patients.

Parameters	MK-4		MK-7	
	r	*p*-Value	r	*p*-Value
Troponin (ng/mL)	−0.68	≤0.001	−0.81	≤0.001
LV ejection fraction (%)	0.61	≤0.001	0.67	≤0.001
Glucose Level (mg/dL)	−0.64	≤0.001	−0.75	≤0.001
Hb (g/dL)	−0.23	0.148	−0.27	0.078
HCT (%)	−0.10	0.212	−0.13	0.150
RBCs (10^6^/uL)	−0.11	0.187	−0.18	0.145
MCV (fL)	−0.12	0.165	−0.13	0.152
MCH (pg)	−0.08	0.242	−0.11	0.180
MCHC (g/dL)	−0.12	0.168	−0.15	≤0.120
Total WBCs (10^3^/uL)	−0.15	0.123	−0.18	0.092
Platelets (10^3^/uL)	−0.35	≤0.01	−0.42	≤0.01
Total Cholesterol (mmol/L)	−0.71	≤0.001	−0.77	≤0.001
HDL-Cholesterol (mmol/L)	0.60	≤0.001	0.69	≤0.001
LDL-Cholesterol (mmol/L)	−0.68	≤0.001	−0.73	≤0.001
Triglycerides (mmol/L)	−0.27	≤0.05	−0.35	≤0.05

r, Pearson’s correlation coefficient.

## Data Availability

Data sharing is applicable to this article.
